# Generation of direct antigen-sandwich enzyme-linked immune-sorbent assays (ELISA) for quantitation of HTLV-1 gp46, p24, Tax and related host cellular antigens OX40, OX40L and CD25

**DOI:** 10.1186/1742-4690-11-S1-P73

**Published:** 2014-01-07

**Authors:** Reiko Tanaka, Yoshiaki Takahashi, Akira Kodama, Mineki Saito, Yuetsu Tanaka

**Affiliations:** 1 Department of Immunology, Graduate School of Medicine, University of the Ryukyus, Okinawa, Japan; 2 RIMCO Ryukyu Immunology Corporation, Okinawa, Japan; 3 Department of Microbiology, Kawasaki Medical School, Kurashiki, Okayama, Japan

## 

A direct antigen-sandwich ELISA using an enzyme-labeled detector antibody has been used as an easy, quick and quantitative assay for many antigens. Generation of such a quantitative and easy ELISA for various HTLV-1 specific antigens and HTLV-1-related host cell antigens will be helpful in various HTLV-1 studies. Thus, we screened our monoclonal antibody (mAb) library to find appropriate combinations of capture and HRP-labeled detector mAbs that enabled quantitation of target antigens. Using our mouse and rat mAbs, we have established direct ELISA kits specific for HTLV-1 core p24, gp46 and Tax, and Tax-induced host antigens including OX40 (CD134), OX40L (CD252) and IL-2R alpha (CD25). These kits were applicable for quantitation of antigens in not only culture supernatants and cell lysates but also human serum/plasma. Preliminary experiments using these kits showed that HTLV-1 p24 and gp46 together with OX40 and CD25, but not Tax or OX40L, were released into culture supernatants of HTLV-1-infected T cells. Interestingly, examination of plasma from various HTLV-1 infected donors showed that soluble OX40 as well as CD25 levels were significantly higher in ATL patients than the others. Soluble OX40L, p24, gp46 and Tax antigens were negative in any plasma tested. We hope that these ELISA kits will be helpful for many fields of studies on HTLV-1 and related diseases.

